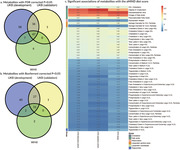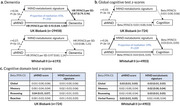# Circulating metabolomic profile of the MIND diet and its relation to cognition in middle‐aged and older adults

**DOI:** 10.1002/alz.088163

**Published:** 2025-01-09

**Authors:** Hui Chen, Jie Shen, Yang Tao, Yaodan Zhang, Mengyan Gao, Yuan Ma, Yan Zheng, Geng Zong, Qing Lin, Lusha Tong, Changzheng Yuan

**Affiliations:** ^1^ School of Public Health, the Second Affiliated Hospital, Zhejiang University School of Medicine, Hangzhou, Zhejiang China; ^2^ Oxford University, Oxford, Oxford United Kingdom; ^3^ Harvard T.H. Chan School of Public Health, Boston, MA USA; ^4^ State Key Laboratory of Genetic Engineering, Human Phenome Institute, and School of Life Sciences, Fudan University, Shanghai China; ^5^ Shanghai Institute of Nutrition and Health, Chinese Academy of Sciences, Shanghai China; ^6^ The Second Affiliated Hospital, Zhejiang University School of Medicine, Hangzhou, Zhejiang China

## Abstract

**Background:**

Dietary factors are modifiable risk factors for dementia. In particular, the Mediterranean‐DASH Intervention for Neurodegenerative Delay (MIND) diet has been associated with better cognitive function and lower risk of dementia. However, circulating metabolomic characteristics of the MIND diet and its associations with cognitive function remains unclear.

**Method:**

In the current study, 45906 UK Biobank (UKB) participants (mean age: 56.4 years) were separated into a discovery cohort and an internal prospective validation cohort, and 6193 Whitehall II (WHII) study participants (mean age: 56.8 years) constituted an external validation cohort. We identified the metabolites associated with the alternate MIND diet score (aMIND) using linear regression models, and Benjamini‐Hochberg method was applied to control false discovery rate. We constructed a metabolomic signature score of the MIND diet using elastic net model and assessed the potential mediating role of the metabolomic signature in the associations of the aMIND with cognitive outcomes in the two cohorts.

**Result:**

The aMIND showed significant associations with 149 out of 168 (89%) metabolites in the UKB discovery cohort, and 47 of these associations were also replicated in both the internal validation cohort and the external validation cohort, including 38 lipoprotein subclass, 4 fatty acid, 1 cholesterol, 1 inflammation, 2 lipoprotein particle size, and 1 triglyceride measures. The metabolomic signature score based on the selected metabolites was significantly correlated with the aMIND (Pearson’s r = 0.36, 0.31, and 0.27 in the discovery, internal validation and external validation cohort). In the UKB (491 incident dementia cases during a median follow‐up of 11.8 years), but not WHII (169 in 17.8 years), the MIND metabolomic signature score significantly mediated the association of the aMIND and incident dementia by 45%. In the WHII, association between the aMIND and cognitive function was partially mediated by the metabolomic signature (proportion = 19%, P for mediation = 0.039).

**Conclusion:**

MIND diet was favourably associated with a panel of metabolites, and a MIND diet metabolomic signature score partially mediated the observed associations between MIND diet adherence and incident dementia and cognitive function.